# Human Genetics to Identify Therapeutic Targets for NAFLD: Challenges and Opportunities

**DOI:** 10.3389/fendo.2021.777075

**Published:** 2021-12-07

**Authors:** Xiaomi Du, Natalie DeForest, Amit R. Majithia

**Affiliations:** ^1^ Division of Endocrinology, Department of Medicine, University of California San Diego, La Jolla, CA, United States; ^2^ Bioinformatics and Systems Biology Graduate Program, University of California San Diego, La Jolla, CA, United States; ^3^ Biomedical Sciences Graduate Program, University of California San Diego, La Jolla, CA, United States

**Keywords:** NAFLD, NASH, human genetics, gene discovery, GWAS, exomes

## Abstract

Non-alcoholic fatty liver disease (NAFLD) is a continuous progression of pathophysiologic stages that is challenging to diagnose due to its inherent heterogeneity and poor standardization across a wide variety of diagnostic measures. NAFLD is heritable, and several loci have been robustly associated with various stages of disease. In the past few years, larger genetic association studies using new methodology have identified novel genes associated with NAFLD, some of which have shown therapeutic promise. This mini-review provides an overview of the heterogeneity in NAFLD phenotypes and diagnostic methods, discusses genetic associations in relation to the specific stages for which they were identified, and offers a perspective on the design of future genetic mapping studies to accelerate therapeutic target identification.

## Introduction

Non-alcoholic fatty liver disease (NAFLD) is the most prevalent liver disease globally, affecting approximately 25% of the adult population as of 2016 (1), and its incidence continues to increase. NAFLD encompasses simple steatosis (fatty liver; NAFL) and the more severe nonalcoholic steatohepatitis (NASH), which is characterized by fat accumulation, inflammation, and hepatocellular injury. Hepatic fibrosis can develop in NAFLD, which can progress into cirrhosis and hepatocellular carcinoma (HCC) (2, 3). As of 2019, NASH was the underlying cause of liver failure in over a third of individuals awaiting liver transplant (4). There are currently no FDA approved treatments for any stage of NAFLD, including NASH (5), highlighting the critical need to identify therapeutic targets.

Given that NAFLD is heritable, with heritability estimates ranging 20%-70% (6), genetic mapping has been undertaken to identify causal genes with potential therapeutic implications. Initial NAFLD studies focused on selected candidate genes, but were limited by small sample size, a high rate of false positive associations due to cryptic population stratification, and reliance on prior knowledge for gene selection (7–9). With the advent of genome-wide association methods that could be applied at population scale, some of these limitations have been overcome, resulting in the unbiased, reproducible genetic discoveries that are detailed below.

In this mini-review, we focus on the phenotypic complexity of NAFLD, the challenges this poses to executing genetic association studies, and the progress made in identifying new putative targets over the past four years.

## NAFLD Definitions and Diagnostics

NAFLD is a continuum of disease with multiple pathophysiologies and is defined and diagnosed by variable, often incompatible, approaches. In this section, we provide an overview of this heterogeneity in pathogenesis and detection, focusing on how this impacts the interpretation of the genetic associations studies described below. This brief summary does not cover the full breadth of this field, so we refer the reader to other recent reviews for a comprehensive treatment of all diagnostic methods (10), noninvasive diagnostic modalities (11–13), biomarkers (14, 15), and elastography techniques (16).

**Figure 1:**
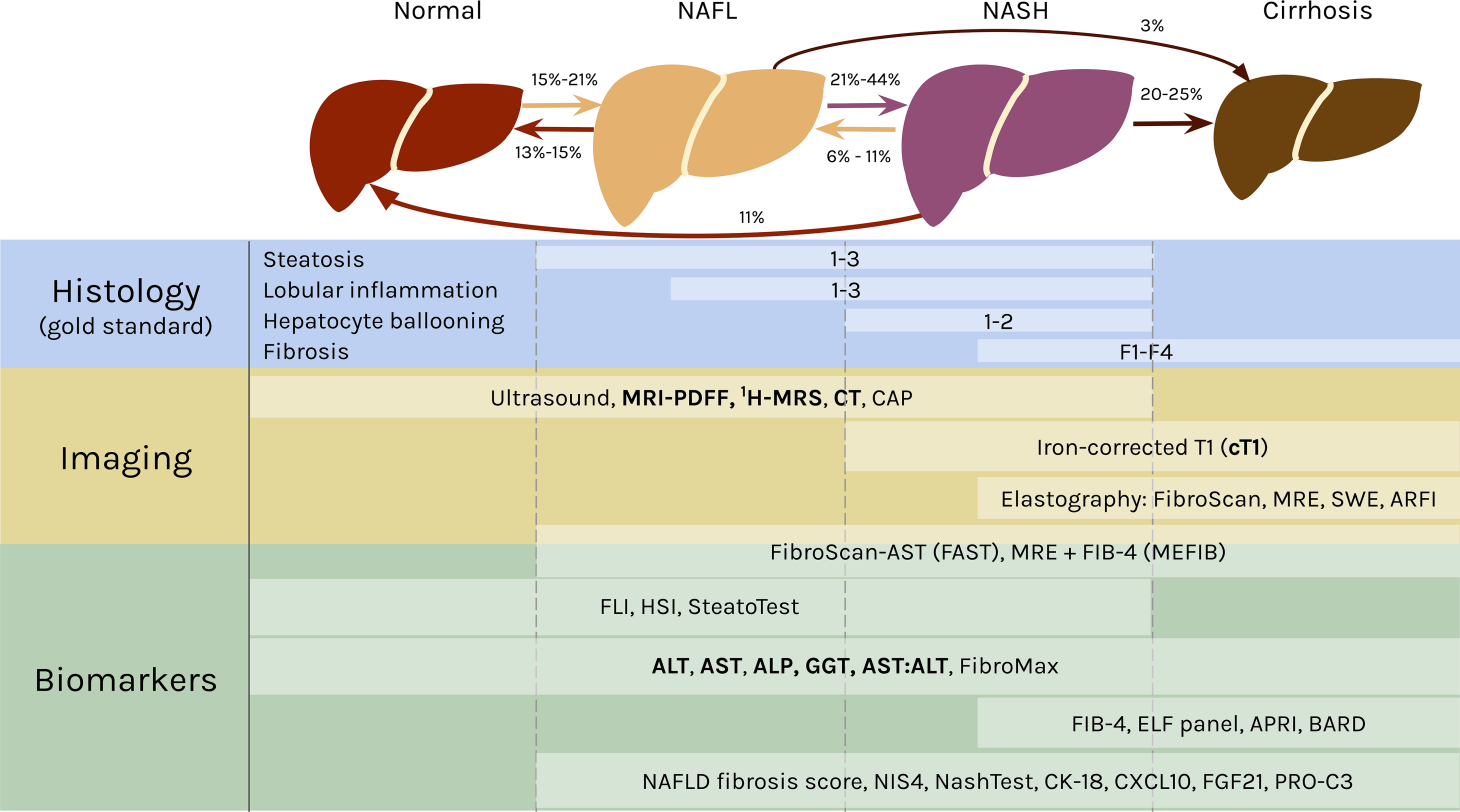
The spectrum of NAFLD and stage specific clinical measures. In NAFL, at least 5% of the hepatocytes have fat accumulation in the form of large lipid droplets in the cells that displace the nuclei (21) or many small lipid droplets (22). NAFL can also include inflammation. The transition to NASH occurs with hepatocellular injury in the form of ballooning and further inflammation. Fibrosis can develop in NASH and advance into cirrhosis, in which the liver shrinks and hardens. NAFL and NASH are reversible, as indicated by the rates regressing in severity. Both can also progress into cirrhosis. The rates of transition between each stage are broad ranges because they originate from studies with varying cohort sizes, time frames, treatments, and other variables (18, 19, 23–26). Histology is the gold standard for diagnosing NAFLD and classifying the stage of disease. The components of NAS are listed above, with the area to the left of the white bars indicating scores of 0 for each feature. A selection of noninvasive diagnostic methods are shown below, with white boxes representing their range in effectively diagnosing different stages of NAFLD [ultrasound (27); FAST (28); MEFIB (29); SteatoTest (30); FibroMax (31); BARD (32); NAFLD fibrosis score (33); NIS4 (34); NASHTest (35)]. The lists are not comprehensive, and the modalities mentioned in the mini-review are emphasized in bold. CAP, controlled attenuation parameter; obtained from FibroScan (36, 37). MRE, magnetic resonance elastography (38). SWE, shear-wave elastography (38). ARFI, acoustic radiation force impulse (37). FIB-4, fibrosis-4 (39). FLI, fatty liver index (40). HSI, hepatic steatosis index (41). ELF, Enhanced Liver Fibrosis (42). APRI, AST-to-platelet ratio index (43). CK-18, cytokeratin 18 (44). CXCL10, C-X-C motif chemokine ligand 10 (45). FGF21, fibroblast growth factor 21 (44). PRO-C3, plasma collagen type III (46).

Clinically, the spectrum of fatty liver disease encapsulated in NAFLD is defined in the absence of excess alcohol intake (5). The distinction between NAFL and NASH is most commonly differentiated by the absence (NAFL) or presence (NASH) of hepatocyte ballooning (17). Some studies further delineate phases between NAFL and NASH (18, 19), and between NASH and cirrhosis (20), highlighting the continuum of pathophysiology. For simplicity, this mini-review will anchor on three stages of NAFLD – NAFL, NASH, and cirrhosis as depicted in **Figure 1**.

### Histology Is the Gold Standard

Liver biopsies are the gold standard for NAFLD diagnosis, and the FDA requires evidence of histologic improvement for NAFLD treatments in late stage clinical trials for consideration of approval (47). In clinical research, biopsies are commonly graded by the NAFLD Activity Score (NAS), which quantifies NAFLD severity based on steatosis (0-3), lobular inflammation (0-3), hepatocyte ballooning (0-2), and fibrosis (0-4) (48) (**Figure 1**). NAS was not designed to be a diagnostic tool, so defining NASH by a cut off threshold of NAS ≥ 5 can result in inaccurate diagnoses (49, 50). There are also different scoring criteria, including the Brunt score (51) and the steatosis-activity-fibrosis (SAF) score (52), that can be used to grade biopsies, and this lack of a single standard leads to difficulties in comparisons between studies. This is further exacerbated by sampling variability due to histologic heterogeneity (53) and subjectivity in interpretation for liver biopsies (54–56). This gold standard based on tissue sampling also limits the investigation of NAFLD at scale for large cohort studies, and biopsies are often refused by patients in clinical practice (15). Thus, there has been a strong emphasis by clinicians and researchers on the development of alternative, noninvasive diagnostic techniques.

### Noninvasive Methods of Diagnosis

There are many imaging methods to detect hepatic steatosis, including computed tomography (CT), magnetic resonance imaging-proton density fat fraction (MRI-PDFF), and proton magnetic resonance spectroscopy (^1^H-MRS) (**Figure 1**). CT can quantitatively measure liver fat content, but it has poor sensitivity for mild steatosis (57), and it exposes patients to ionizing radiation (58, 59). MRI-PDFF and ^1^H-MRS both measure steatosis with high accuracy relative to histologic references, even at low amounts of hepatic fat, so they are the recommended imaging modalities for liver fat (60–62).

Although these imaging methods can accurately measure steatosis, they are poorly suited to detect the features differentiating NASH from NAFL, i.e., ballooning, inflammation, and fibrosis (63). A recently published protocol for multiparametric magnetic resonance (MR) has bridged that gap. MR derived iron-corrected T1 (cT1) is a novel noninvasive method to assess fibrosis (64), and it correlates with all the histological features of NASH (65, 66).

Liver enzyme levels have also been correlated with NASH and fibrosis (67). The classic indications of liver inflammation are aspartate aminotransferase (AST), alanine aminotransferase (ALT), gamma-glutamyltransferase (GGT), and alkaline phosphatase (ALP), along with the AST/ALT ratio (68, 69) (**Figure 1**). Elevated enzyme levels are insufficient to provide a confident NAFLD diagnosis, however, because ALT values are normal in up to 25% of NAFLD patients (70, 71).

Altogether, no single noninvasive method has replaced histology yet for detection of all the phenotypes characteristic of NAFLD. Nevertheless, studies have effectively employed combinations of these alternative modalities to measure the full spectrum of NAFLD features.

## Genetic Approaches for NAFLD Therapeutic Target Identification

Epidemiological, familial aggregation, and twin studies over the past two decades have demonstrated a heritable component to NAFLD (72), strongly suggesting that genetic mapping approaches could be productively deployed to identify genes with therapeutic potential. As mentioned earlier, initial genetic investigations into NAFLD utilized candidate gene approaches, but the development of next-generation sequencing (NGS) and high-throughput genotyping arrays enabled more robust, unbiased methods of genetic mapping studies including genome-wide association studies (GWAS) and exome-wide association studies (EWAS) (73, 74). GWAS has successfully identified loci that are associated with risk for many complex diseases and traits using common variants ascertained from genotyping (75), whereas EWAS examines variants predominantly in the exonic (i.e. protein-coding) regions of the genome (76). With the decreasing cost of NGS, current studies can detect exonic variants through whole-exome sequencing (WES) (77). Recent expert reviews have summarized variants identified from NAFLD genetic association studies (6, 78, 79). Here, we build upon these publications by reviewing the literature from the past four years, highlighting the consequence of NAFLD phenotypic heterogeneity on genetic discovery, and quantifying the limits of current association studies to identify new genetic signals.

### Genetic Associations Discovered in the Past Four Years

We focus our attention on novel loci discovered in NAFLD related GWAS and EWAS from the past four years.

Abul-Husn et al. performed an EWAS for ALT and AST levels using WES (n=46,544) and validated their associations in two additional cohorts (n=9,883) and liver biopsy samples (n=2,391) (80). They found that a loss of function, protein-truncating variant in *HSD17B13* (rs72613567:TA) was associated with decreased levels of ALT and AST and lower rates of NASH, as determined by the presence of any inflammation or hepatocyte ballooning in liver histology. At the same time, this variant was not associated with NAFL (80), providing evidence that *HSD17B13* may be involved in more clinically advanced stages of NAFLD.

Namjou at el. used a natural language processing (NLP) algorithm to identify NAFLD cases for a GWAS in pediatric and adult cohorts (1,106 cases and 8,571 controls) (81). They replicated associations between NAFLD and variants in the *PNPLA3-SAMM50-PARVB* locus (including rs738409). Namjou et al. subsequently performed quantitative case-only association studies for NAS, fibrosis, AST and ALT, finding that *IL17RA* was associated with NAS, and *ZFP90-CDH1* was associated with fibrosis.

Anstee et al. conducted the largest GWAS to date for NAFLD ascertained by histology (1,483 cases and 17,781 controls) and identified two new associations (82). An intronic variant near the *LEPR* gene was associated with NASH at genome-wide significance, and a missense variant in *PYGO1* encoding p.P299H (rs11858624) was associated with protection from steatosis at close to genome-wide significance (82).

Parisinos et al. performed a GWAS for liver inflammation and fibrosis using cT1 values (n=14,440) and studied the associations between significant variants and liver biomarkers (n=378,821). Novel variants in *SLC30A10* and *SLC39A8* had genome-wide significant associations with cT1 and elevated levels of ALT and AST. In a separate GWAS performed on the same cohort (n=14,440), four variants were associated with steatosis measured by MRI-PDFF, including *APOE* rs429358, a missense variant that encodes p.C112R. Parisinos et al. further studied the associations between cT1 values and variants identified by a cirrhosis GWAS, which found a missense variant in *MARC1* encoding p.A165T (rs2642438) that protects against cirrhosis (83). This analysis revealed that variants in *MARC1* and *HSD17B13* were associated with both cirrhosis and cT1 values (84).

A recent study of protein-coding variants ascertained by genotyping arrays investigated genetic associations for ALT levels (n=425,671) (85). The authors found 190 genetic variants associated with ALT, replicated their findings in three public GWAS databases, and associated the variants with liver fat as measured by MRI-PDFF (n=8,930) to validate significant variants. These variants, including single nucleotide polymorphisms (SNPs) in *MARC1*, *APOE*, and *GPAM*, were all additionally associated with chronic liver disease and cirrhosis. Jamialahmadi et al. further validated these genetic associations with liver biopsies (n=2,621). The missense variant in *GPAM* (rs2792751) encoding p.V43I was found to be significantly associated with severity of liver steatosis, while *APOE* rs429358 confers protection for liver steatosis. The association between *APOE* and NAFLD was also found in an exome-wide association meta-analysis of CT-measured liver steatosis across eight multi-ethnic population-based cohorts (n=16,492) (86).

Pazoki et al. performed GWAS on serum levels of ALT, ALP, and GGT (n=437,438) and replicated their results in three additional cohorts (n=315,572) (87). These enzymatic indicators of inflammation and liver disease were associated with 517 SNPs, including variants in *SERPINA1*, *APOE, GPAM, MARC1*, and *LEPR*. The number of variants associated with any combination of ALT, ALP, and GGT is likely greater than the number found by studies that used imaging or histology to assess NAFLD because serum levels are not specific to NAFLD and are reflective of many processes in the body, including cardiovascular disease (87).

Liu et al. applied deep learning to MRI scans to quantify volume, fat, and iron in many organs, including the liver (n=38,881), and performed GWAS on their results (n=32,858 for liver fat). Variants near *PPP1R3B* and in *GCKR* were associated with liver volume, which was strongly correlated with liver function (88). Liu et al. also identified eight variants associated with liver fat, including *TRIB1* rs112875651, *MARC1* rs2642438, *GPAM* rs11446981, and a region in *MTTP*.

### Genetic Associations in the Context of NAFLD Phenotypic and Diagnostic Heterogeneity

**Figure 2:**
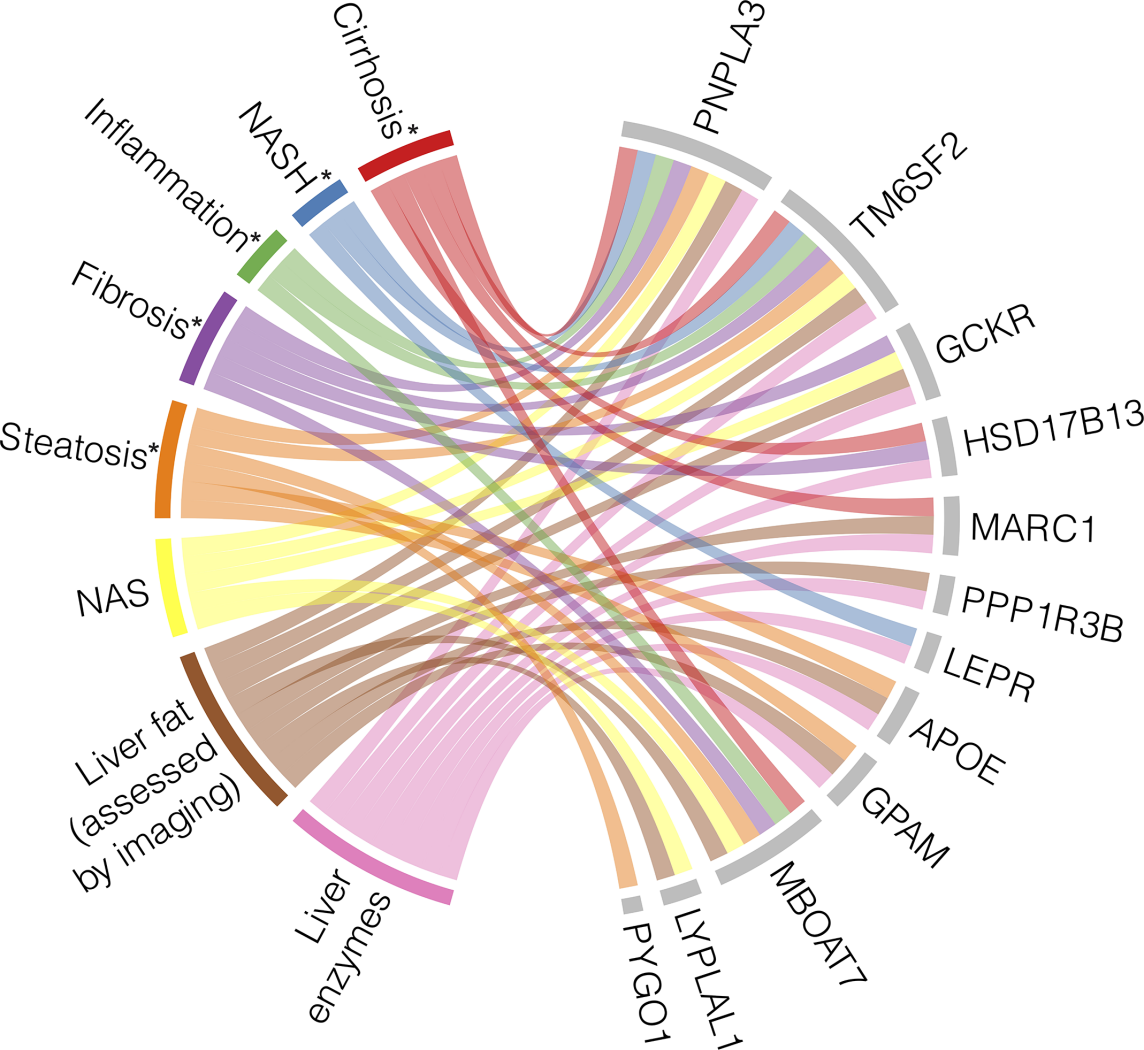
Heterogeneity and pleiotropy of genes associated with NAFLD phenotypes. Loci with their corresponding genes which have been shown to be associated with NAFLD through genome- and/or exome-wide association studies are displayed on the right side of the chord diagram, indicated by grey bars. On the left side of the diagram are the NAFLD states with their associated measurements, each represented by a different color. Each ribbon represents a significant association identified between each gene and the described state. * indicates histologically defined state (56, 80, 82, 84–87, 89–96).

Multiple GWAS and EWAS have been conducted to find genetic associations with specific features of NAFLD, such as hepatic steatosis, fibrosis, and liver inflammation, as well as the full spectrum of disease. Some variants have been associated with the full NAFLD spectrum, while others are only correlated with certain phenotypes. The specific NAFLD phenotypes and measurements that these genes have been associated with through GWAS and/or EWAS are summarized in **Figure 2**.

A missense SNP in *PNPLA3* (rs738409) encoding p.I148M is the most robustly associated genetic variant with the full spectrum of NAFLD (78). The landmark *PNPLA3* study measured hepatic fat content by ^1^H-MRS and examined inflammation through serum levels of ALT to suggest that rs738409 could increase risk of NASH, but the study itself did not focus on histologic NASH or cirrhosis (97). Since then, many GWAS and EWAS have identified a relationship between *PNPLA3* and steatosis measured by other imaging methods (86), histologically defined steatosis, hepatocyte ballooning, lobular inflammation, fibrosis, and cirrhosis (82, 89) and cT1 defined NASH (84).

Similarly, the missense variant rs58542926 encoding p.E167K in *TM6SF2* was initially associated with hepatic fat measured by ^1^H-MRS and liver enzyme levels (90), and it has since been found to associate with the full range of NAFLD phenotypes. *TM6SF2* rs58542926 has been robustly associated with steatosis assessed by CT in independent studies (84, 86). The initial AST, ALT, and ALP associations were replicated by Parisinos et al. and further supported by associations between the variant and cT1 values (84) and histologically ascertained NAS and SAF scores (91), indicating that *TM6SF2* rs58542926 is implicated in NASH in addition to NAFL. *TM6SF2* was also associated with histologically graded cirrhosis (84).

Several other loci have been pleiotropically associated with multiple NAFLD stages. *GCKR* rs1260326 is associated with hepatic steatosis, as assessed by both imaging and histology (84, 86, 92, 93), inflammation measured by serum enzyme levels (87, 92), histological assessments of NAFLD graded by NAS (94), histological fibrosis (82), and overall liver function (88). *MBOAT7* has similarly been examined across the entire spectrum of NAFLD, ranging from liver fat accumulation to cirrhosis (85, 95, 98), but intriguingly, it was found to be independently associated with fibrosis development in particular, suggesting a unique molecular mechanism (56). Finally, *MARC1* has been associated with steatosis (85, 88), inflammation/NASH (84, 85, 87), and cirrhosis (83, 84).

Conversely, some genetic associations have been identified for only specific NAFLD stages and diagnostic modalities. As described above, *PYGO1* has only been associated with histologically identified steatosis (82). *GPAM*, *PPP1R3B*, and *APOE* have associations with steatosis (84–86, 88, 93, 94) and serum enzyme levels (85, 87, 92), but these loci have not been associated with histological features of NASH or cirrhosis. On the other hand, *LEPR* is only associated with ALT levels and histologically defined NASH (82, 87), and *HSD17B13* is associated with NASH and cirrhosis (80, 84).

There are some variants that have only been identified in a single study so far, introducing uncertainty in their relationship with NAFLD. For example, variant rs12137855 mapped to *LYPLAL1* has been associated with liver fat and histologic NAFLD, as quantified by NAS (94), but this SNP has not been replicated in this past decade by other association studies. A possible explanation for this lack of reproducibility is the combination of the small effect size of the *LYPLAL1* variant and the current limits in statistical power.

## Current Study Design Limitations to Discovering NAFLD Associated Variants

The variants identified through GWAS and EWAS are susceptible to the study design choices. The sample size, diversity within the cohort, and specificity of the associated trait, along with many other confounders, can all affect the results (99). A major cofounder is that sample size in current NAFLD studies is highly correlated to the measurement modality. On the spectrum of sample numbers, liver enzyme levels, which are commonly available as part of routine blood testing, are on the high end, and liver biopsies, which require a clinical indication and are difficult to perform in large numbers, are on the low end (63). Because statistical power to detect significant associations is directly dependent on sample size (100), studies using biopsies are often underpowered, while studies using serum concentrations are better powered but less informative for NAFLD stages.

**Figure 3:**
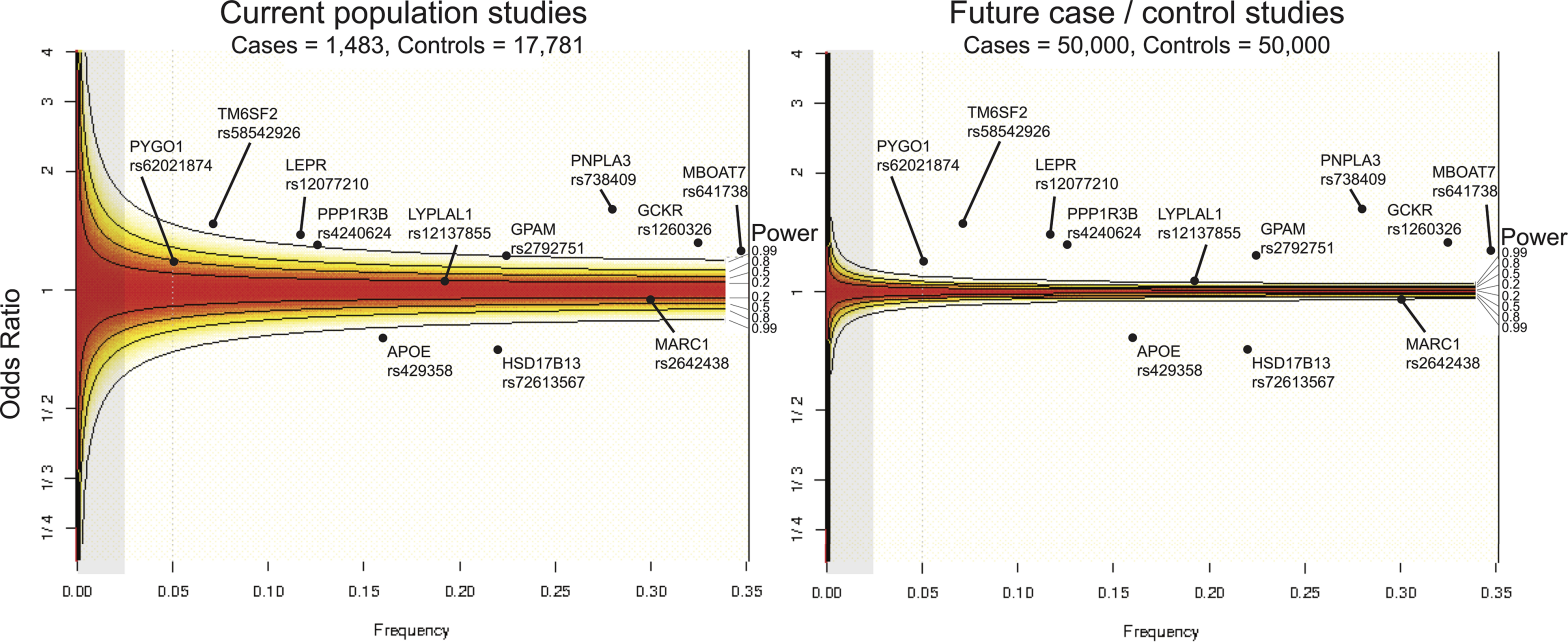
Current state of statistical power to detect genetic associations with NAFLD. Statistical power across odds ratios per allele frequencies computed with a type 1 error rate set at α=0.05 shown for current sample size of NAFLD population studies (left) and for an idealized future NAFLD case/control study (right). Power for frequencies and odds ratios regions are shaded from red to white, in which red indicates regions where statistical power is not sufficient to detect an association, and white areas are within the detection limit. Gray areas highlight the section of the power curve for associating rare genetic variants (MAF < 0.025). Associated loci are overlaid according to their effect size and minor allele frequencies. For example, *PNPLA3* rs62021874, which has been robusted associated with NAFLD across several studies, is well within the detectable region given its minor allele frequency of 0.28 and odds ratio of 1.8, whereas *PYGO1* (frequency = 0.05, odds ratio = 1.3), which has been associated in a single study, is nominally powered to detect an association given the current sample size of NAFLD population studies (56, 82, 85, 93).

In **Figure 3** (left panel), we illustrate the statistical power of the largest liver biopsy GWAS to date (82), which included 1,483 NAFLD biopsied cases and 17,781 controls. Given its size, this study would be predicted to successfully replicate previously characterized loci, including *PNPLA3, TM6SF2, HSD17B13*, and *GCKR*, based on their respective frequencies in the population and effect sizes on NAFLD risk. These associations are indeed found with genome-wide significance (82). Furthermore, a GWAS of this size would be predicted to not detect *MARC1* and *LYPLAL1* with genome-wide significance, as those variants have smaller effect sizes. Again, this is reflected in the results: although the variant in *MARC1* was associated with NAFLD with *p* < 6 x 10^-6^, the association did not meet the genome-wide significance threshold (82).

Future studies with increased sample sizes of NAFLD individuals and balanced case/control designed studies may reveal novel genetic associations which studies are currently underpowered to detect and provide additional support for existing associations (**Figure 3**, right panel). With the generation of larger NAFLD case/control cohorts and increased application of WES, more rare variants with large biological effects can be identified, which would facilitate therapeutic targeting. Rare, loss-of-function variants that confer protection from disease in particular have shown promise as therapeutic targets, as exemplified by the successful development of *PCSK9* inhibitors to treat atherogenic cardiovascular disease (101, 102).

## Current Perspective on NAFLD Association Studies

Identifying causal genes is a major challenge to translating genetic association signals into biological and potentially therapeutic knowledge. The majority of variants identified from GWAS are located in non-coding genomic sequences distant from protein-coding genes (99). For example, a variant on chromosome 8 that lies in the intergenic region between *IDO2* and *TC1* was associated with NAFLD, but it is unclear which gene is driving the phenotype (82). Additionally, while it is standard practice to designate the nearest gene to a variant as the causal gene, this may not always be true. This caveat was showcased by the variant rs2075650 residing in an intron of *TOMM40*, which was found to be associated with steatosis. While most proximal to the *TOMM40* coding sequences, conditional analysis showed that this variant association was driven by linkage disequilibrium with the previously identified *APOE* rs429358 (86). In contrast to GWAS, EWAS analyses almost entirely use variants in the exonic regions of the genome, which can make causal gene identification more straightforward as the variants likely alter the sequence of the encoded protein. However, to date, the findings of NAFLD EWAS are still limited. So far, only one study specific to NAFLD has harnessed rare protein-coding genetic variants from WES rather than genome- or exome- arrays (103), but additional large-scale studies of rare variants and their effects on NAFLD are beginning to emerge and find new signals such as *MAST3* and *IFI30* (104, 105).

As mentioned earlier, increasing sample size to power robust discovery is a current challenge in NAFLD gene discovery due in large part to inherent limitations in the scalability of liver biopsies. Some studies have employed creative methods to increase sample sizes for their NAFLD genetic studies with some indications of success. These techniques include NLP algorithms (81), machine learning applied to liver imaging (88, 104), and a multi-step approach of first identifying genetic signals with a widely available biomarker, such as liver enzyme levels, in population cohorts, and then examining only these identified signals with independent histological cohorts (80, 94) to decrease the multiple hypothesis correction burden. For example, Abul-Husn et al. first conducted a GWAS in almost 47,000 individuals to identify variants significantly associated with either ALT or AST levels (80). 13 of these variants were next replicated in an additional cohort (n=12,527), and then these top variants were identified within exome sequences (n=1,857 NAFLD cases and 29,928 controls) and tested for association with chronic liver disease. This reduced the statistical threshold for significance without increasing the false positive rate. From this targeted exome association analysis, the protein-truncating variant in *HSD17B13* (rs72613567) was found to confer lower odds across all categories of liver disease and provide protection against liver fibrosis in an allele dose-dependent manner. This discovery then led to the development of ARO-HSD, a RNAi therapeutic that selectively targets *HSD17B13* mRNA in hepatocytes, which has demonstrated improvements in NASH outcomes, as assessed by ALT, AST, and MRI-PDFF, in a Phase 1/2 clinical trial (106, 107).

In the serendipitous case of *HSD17B13*, the consequence of the top identified variant was protein-truncating and thus could be predicted with high confidence to confer loss of function in HSD17B13 without additional functional characterization. In order to provide analogous interpretations to genetic variants that do not have such clear cut functional effects without performing validation experiments, NAFLD genetic studies have utilized computational prediction tools (108), ClinVar reported pathogenicity predictions, and allele frequency cut-offs (109), to narrow the search space to actionable variants, but with limited success in the absence of mechanistic investigation. In summary, despite current limitations, genetic discoveries for NAFLD have demonstrated promise in therapeutic target identification. Future genetic investigations with increased sample size and focusing on different stages of NAFLD are likely to reveal new genes with therapeutic potential.

## Future Directions

Efforts are underway to improve standardization in classification and diagnosis of NAFLD to enable translational research that can identify putative drug targets. In 2020, an expert consensus panel proposed a new set of diagnostic criteria for NAFLD (110) and renamed it metabolic associated fatty liver disease (MAFLD). MAFLD is diagnosed by the presence of hepatic steatosis (ascertained by imaging, biomarker panel or histology) and either type 2 diabetes (T2D) or overweight/obesity, or two of the following metabolic risk factors: waist circumference, blood pressure, serum triglycerides, low serum HDL, prediabetes, insulin resistance, and plasma high-sensitivity C-reactive protein level (111). Recent publications indicate that the MAFLD criteria performs better than the NAFLD definition at identifying patients with more severe presentations of disease (112–114), but the new terminology is still heavily debated (115). To date, one genetic association study has been performed using the MAFLD definition and recapitulated the known genetic associations with *PNPLA3* rs738409 and *TM6SF2* rs8542926 (116). This is promising as MAFLD diagnosis does not require biopsies and can be diagnosed from readily available clinical measurements. Nevertheless, further validation is required, especially for the SNPs associated with hepatic injury and fibrosis, which are not explicitly included in the MAFLD definition.

Other than the RNAi targeting *HSD17B13* mRNA mentioned above, most therapeutics currently in clinical trials do not target genes identified from GWAS (63). One possible direction to identify novel, actionable targets for NAFLD from gene or exome wide associations would entail a combination of imaging and biomarkers for NAFLD diagnostic staging that could be broadly applied to hundreds of thousands of individuals in biobanks, as demonstrated by recent publications (84, 85). A specific pathophysiology of NAFLD, such as NASH defined by MRI-PDFF and cT1, should be selected to ensure that there is a sufficiently large cohort of cases for a well-powered study. Association analysis could then be performed to identify rare variants with large effect sizes associated with this classification of NASH. The variants could be further investigated by functional validation in molecular assays to find the causal genes, which would then be the targets of drug development.

## Author Contributions

XD and AM conceived the manuscript outline and figure concepts. XD and ND authored sections of the manuscript and created figures. XD, ND, and AM were involved in critical manuscript revision. All authors contributed to the article and approved the submitted version.

## Funding

This work was supported by grants from the National Institute of Diabetes and Digestive and Kidney Diseases (1R03DK113328-01 and 1R01DK123422-01 to ARM), a UCSD/UCLA Pilot and Feasibility grant (P30 DK063491 to ARM), and a Ruth L. Kirschstein Institutional National Research Service Award T32 GM008666 from the National Institute of General Medical Sciences (to ND).

## Conflict of Interest

The authors declare that the research was conducted in the absence of any commercial or financial relationships that could be construed as a potential conflict of interest.

## Publisher’s Note

All claims expressed in this article are solely those of the authors and do not necessarily represent those of their affiliated organizations, or those of the publisher, the editors and the reviewers. Any product that may be evaluated in this article, or claim that may be made by its manufacturer, is not guaranteed or endorsed by the publisher.
